# Supplementary material for: Contraction of Online Response to Major Events
Source: PLoS One. 2014 Feb 26;9(2):e89052. doi: 10.1371/journal.pone.0089052 (PMC3935844; doi:10.1371/journal.pone.0089052)
Supplement: File S1 — Supporting Information. Figure S1, Characteristic of data set 1 with a one hour timestep. Figure S2, Characteristics of data set 2a: Forum thread during the presidential election night. Figure S3, Characteristics of data set 2b: Forum thread one week before the presidential election night. Figure S4, Characteristics of data set 3: Enron email corpus. Figure S5, Characteristics of data set 4a: Twitter snow storm. Figure S6, Characteristics of data set 4b: Facebook snow storm. Figure S7, Characteristics of data set 5: App.net. Figure S8, Length distribution of all the tweets in data set 1, and corresponding fits of various distributions. Figure S9, Length distribution of all the posts in data set 2a, and corresponding fits of various distributions. Figure S10, Detail from Fig. S9 showing the tail of the distribution. Figure S11, Dependence of the lognormal fitted parameters with the volume. Figure S12, Analysis of the `ideal' subset of tweets. Figure S13, Characteristic of data set 1 with a 5 min timestep. Figure S14, Characteristic of data set 1 with a 3 hours timestep. Table S1, Best fit MSEs () for the tested distributions for the Twitter data set 1. Table S2, Best fit MSEs () for the tested distributions for the forum data set 2a. Table S3, Characteristic parameters measured on data set 1 for different timesteps. (PDF) [file pone.0089052.s001.pdf]

Contraction of online response to major events

Supporting Information

Michael Szell, Sebastian Grauwin, Carlo Ratti

This document is the supporting information to the article ‘Contraction of online response to major events’.

CONTENTS

S1. Data collection and processing 2
Data set 1: Golf Masters tournament on Twitter 2
Data sets 2a and 2b: Presidential election on an online forum 2
Data set 3: Enron email corpus 3
Data sets 4a and 4b: Snow storm “Nemo” in Twitter and Facebook 3
Data set 5: App.net 3

S2. Results from all datasets 4

S3. Fitting of content length distributions 11
Data set 1 11
Data set 2a 13

S4. Influence of different parameters 15

S5. Influence of time step 18

References 21

S1. DATA COLLECTION AND PROCESSING

We have collected several data sets from different online media to check for generality of our results, i.e. independence from the medium. Also, different data sets were collected during times of low excitation and for different time scales to check possible influences from these features. The main text elaborates the findings using data set 1, the similarity of results from other data sets as well as deviations are reported in SI Section S2. The following subsections elaborate the data collection and cleaning process for all data sets.

Data set 1: Golf Masters tournament on Twitter

The first data set was provided by the streaming service Datasift (www.datasift.com) and includes 411,239 tweets and 118,936 retweets between April 5th 00:00:00 EST and April 19th 00:00:00 EST where the following golf-related hashtags have been applied for selection: #golf, #masters, #augusta, #PGA, #themasters, #usmasters. The data set contains a random corpus of 1% of the whole tweet stream during that time. The date range was selected because it overlaps with the 2012 Masters Tournament, one of golf's four major championships, held from April 5–8, 2012, at Augusta, Georgia, United States, organized by the PGA Tour (named after PGA, the Professional Golfers' Association of America). The golfer Bubba Watson won the tournament on April 8th by defeating his opponent Louis Oosthuizen in a sudden-death playoff, which marks the finale of the event. The tweets are generated by 196,386 unique users, all tweets and retweets are generated by 262,165 unique users. On average, each user generates approximately 2 tweets or retweets during the considered time frame. In our analysis we only use tweets and discard retweets as they do not provide original content. Data was provided by Datasift cleaned for duplicates, we have no reports about other cleaning processes, of data loss, or of extraordinary procedures during the collection process. Possible biases towards more central users [1] have not been taken into account. We checked that the vast majority of tweets relates indeed to the golf event and not to identically named topics such as the VW Golf car.

Data sets 2a and 2b: Presidential election on an online forum

We crawled several threads of a popular internet forum, the Something Awful (SA) forums (forums.somethingawful.com). The SA forums have currently a total user base of over 170,000, each day several thousand users are logged in during peak times. The total number of threads that have been posted so far is over 6 million containing over 200 million posts. The SA forums are divided into main forums and several discussion and "finer arts" forums and sub forums, in which users debate specific topics or comment on live events. Threads have a linear structure, but quoting (referring to) previous posts or external sources is possible anytime. Every thread has a unique thread id, accessing a non-archived thread is possible via *showthread.php* and the GET parameter *threadid*, e.g. the URL
http://forums.somethingawful.com/showthread.php?threadid=12345
displays the thread with thread id 12345. The collection process stored contents and timestamps of all thread posts, excluding posts by the forum's ad bot, after applying an inversion of the forum's curse filter on certain curse words and replacing all line breaks with white spaces, image and video links and post attachments with "IMG" and "VID", and all quoted posts with the letters "QUT". The curse filter and ad bot posts affect unregistered users including our crawler.

We crawled a high-volume thread in the *Debate & Discussion* forum, thread id 3515863, titled (ironically) "2012 US Election: Congratulations, President Romney", in which 2425 unique users have debated and commented live on the events unfolding during the US presidential election night of 2012, with a total of 19,019 posts within a time span of almost 60 hours between Nov 6th 2012 01:08 EST and Nov 8th 2012 13:14 EST. The content of this thread constitutes data set 2a, its climax is marked by the confirmation of Barack Obama winning the election (beyond reasonable doubt) shortly before midnight. To compare this high volume event with content from the same medium, and on the same topic, but during a time of lower excitation, we also crawled thread 3514171, data set 2b, which is the predecessor thread to the election night thread, taking place in the week before, with 10,696 posts between Oct 28th 2012 11:31 EST and Nov 6th 2012 01:20 EST performed by 1277 unique users. This thread is more of conversational nature with a smaller rate of live event comments.

Data set 3: Enron email corpus

Certainly the most famous corpus of emails available for study, the Enron Corpus is a large database of over 600,000 emails generated by 158 employees of the Enron Corporation. This database was originally made public by the Federal Energy Regulatory Commission during its investigation of the Enron corporation. The raw corpus contained a lot of duplicate and corrupted emails, several teams have spent time cleaning the data. We used Shetty & Adibi's cleaned dataset [2] available at `http://sgi.nu/enron/corpora.php`. Out of the $\approx 250,000$ emails of their database, we extracted those with original content by removing the 'forward' emails and the 'reply' emails. The forward and reply emails usually contain an original message plus one or more reply messages and were simply identified by the RE: and FW: mentions in the emails' subjects. Dataset 3 hence contains $154,003$ emails generated between Oct 30th 1998 and July 19th 2002.

Data sets 4a and 4b: Snow storm "Nemo" in Twitter and Facebook

Data sets 4a and 4b were obtained through the streaming service Datasift (www.datasift.com) and includes 219,866 tweets and 43,516 Facebook messages written between Thursday, February 7th 12:00 EST and Monday, February 11th 12:00 EST. The snow storm which struck the North-eastern coasts of US and Canada mostly on Friday, Feb 8th, and Saturday, Feb 9th, was dubbed "Nemo" by some weather channel around noon on Thursday, a nickname which became an instant hit on social networks. Data set 4a was extracted by selecting tweets using either the hashtags #nemo or #snowstorm (case insensitive) and data set 4b by selecting Facebook posts containing either the strings 'nemo' or 'snowstorm' (case insensitive). We checked manually that the vast majority of posts relates indeed to the snow storm event and not to identically named topics. Data was provided by Datasift cleaned for duplicates, we have no reports about other cleaning processes, of data loss, or of extraordinary procedures during the collection process. Possible biases towards more central users [1] have not been taken into account. Unlike the data sets 1 and 2a, the snow storm data set does not contain any temporally singular events where the activity on the social network is concentrated on a single point in time (like the "sudden death" golf tournament finale or announcements of Barack Obama winning states or the presidency). Nemo lasted for approximately 48 hours during which the rate of messages stayed rather constant during the day, and slowed down during the night.

Data set 5: App.net

App.net is a subscription based, crowd funded, ad-free microblogging and networking platform very similar to Twitter, aimed at app developers to build on its platform (currently the service is in alpha version: https://alpha.app.net/). Posts in app.net have a character length limitation of 256, which is 116 characters more than Twitter. We crawled the global app.net stream (https://alpha.app.net/global/) and downloaded all posts that have been posted in 181 days between Fri, Aug 3rd 2012 12:59:06 EST, the beginning of the existence of app.net, and Thu, Jan 31st 2013 12:45:17 EST. This data set 5 contains 2,707,158 posts. Out of those, we only consider the 2,574,813 posts which contain content, i.e. which have not been deleted by their posters. These posts are associated to 21,814 unique users. In contrast to Twitter, the service is relatively new, subscription based, and aimed at a particular target audience – these are possible reasons why it is also much less popular. Posts on app.net typically relate to technological news or developments, or are of conversational nature.

S2. RESULTS FROM ALL DATASETS

Here we present the analysis of all data sets performed in the same way as the analysis of data set 1 in the main text. Their general properties are reported in Table 1 of the main text. Number of messages and time ranges span different orders of magnitude.

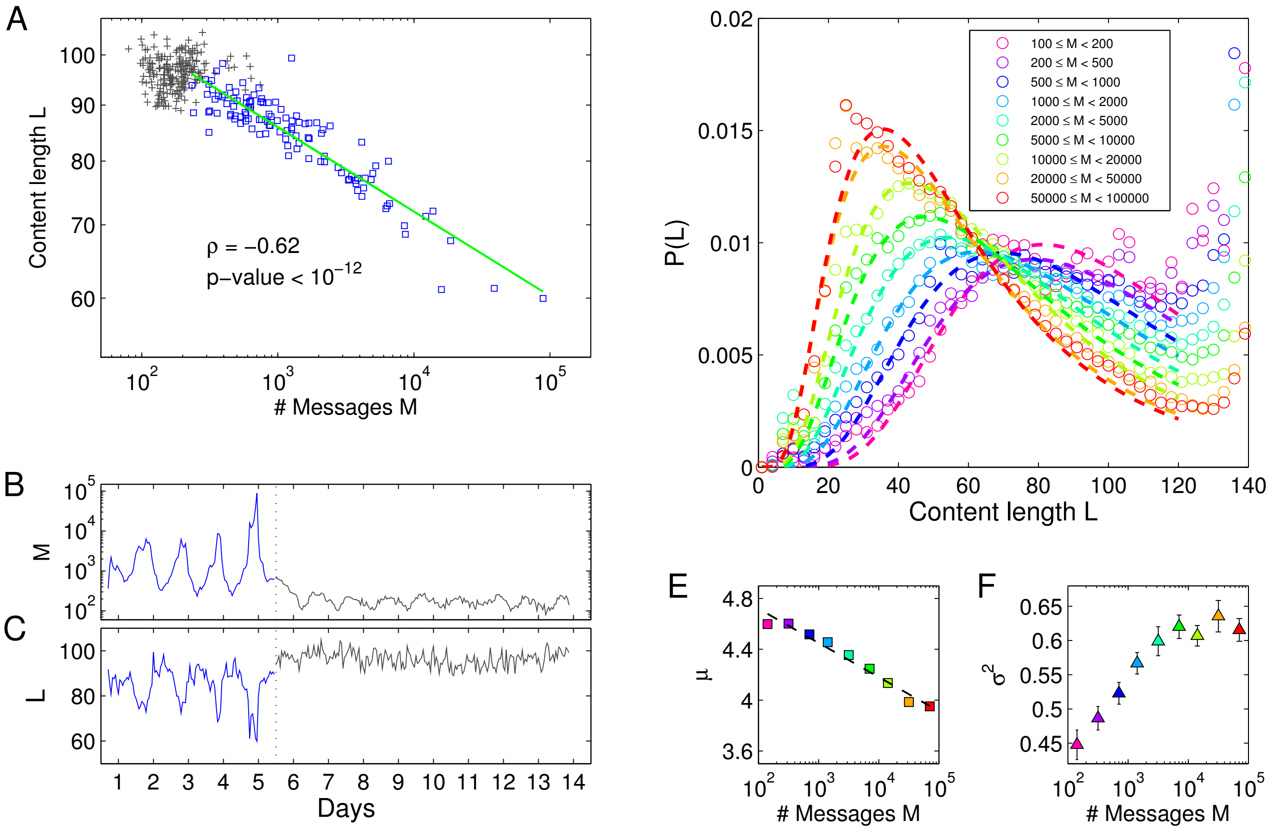

Figure S1. Characteristic of data set 1 with a one hour timestep. (A) The microscopic property of content length L (number of average characters per message) can be related to the macroscopic property of volume (number of messages M) via power law with slope -0.077 (green line), a logarithmic fit cannot be rejected as well due to the flat slope. Blue squares are average hourly values from the first five days in which the Masters Tournament took place, grey crosses are the average values from subsequent times. Volume and content length are strongly anti-correlated ($\rho = -0.62$, p-value $< 10^{-12}$ for the hypothesis of no correlation) but not afterwards ($\rho = 0.04$, p-value $= 0.59$). (B) Respective volume and (C) content length over time. (D) Probability distributions of content length L of messages gathered by classes of hourly volume (circles), and corresponding lognormal fits (dashed curves, fit ranges 0 to 120). For visual clarity an average moving filter of length 5 was applied and only every fifth data point is shown. Fits were performed on the full raw data. (E) Plot of the lognormal fits parameter μ against volume demonstrates the systematic relation between volume and length, dashed line. (F) Plot of the lognormal fits parameter σ^2 against volume.

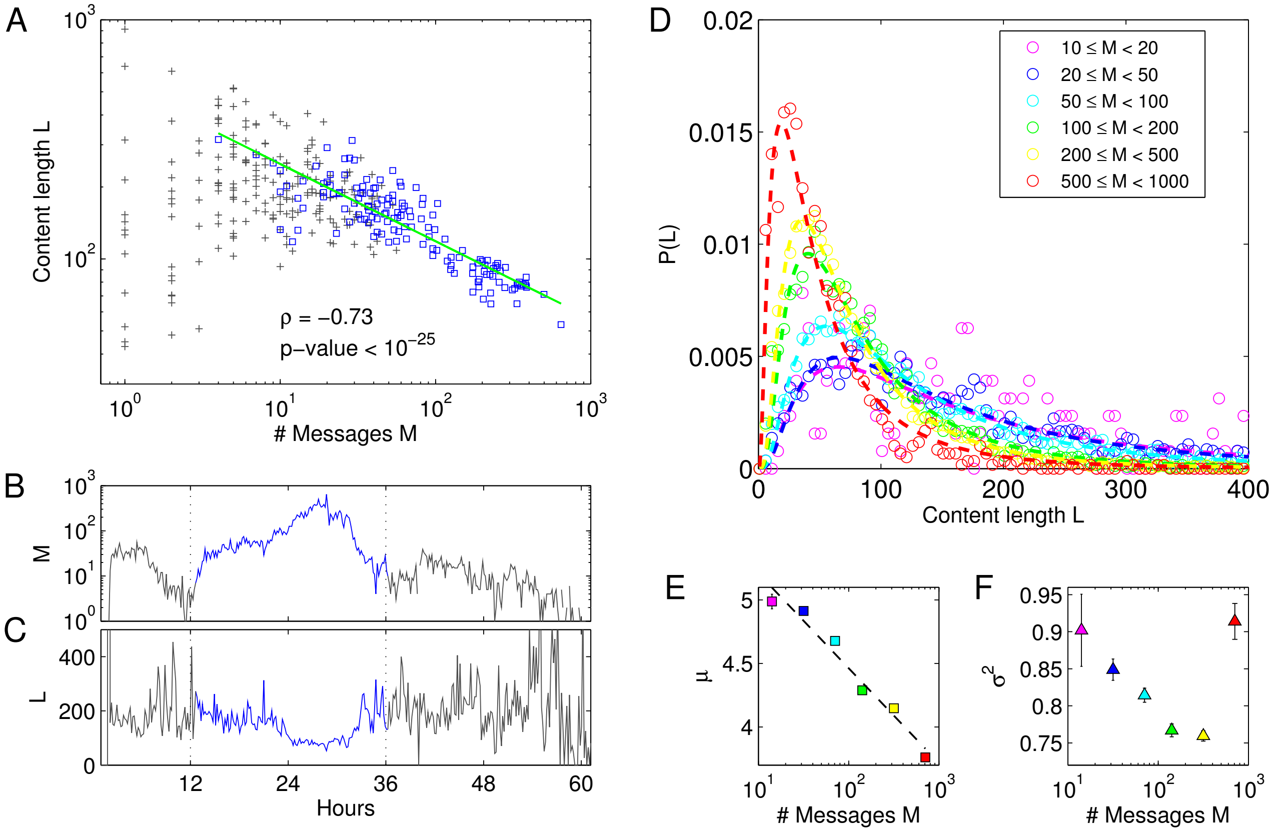

Figure S2. Characteristics of data set 2a: Forum thread during the presidential election night. (A) The microscopic property of content length L (number of average characters per message) can be related to the macroscopic property of volume (number of messages M) via power law with slope -0.32 (green line), a logarithmic fit cannot be rejected as well due to the flat slope. Blue squares are 10 min-aggregated values from the 24 hours of the presidential election night, grey crosses are the values from previous and subsequent times. Volume and content length are strongly anti-correlated during the night ($\rho = -0.73$, p-value $< 10^{-25}$ for the hypothesis of no correlation) but not before and afterwards ($\rho = -0.06$, p-value $= 0.31$). (B) Respective 10 min aggregated volume and (C) content length over time. (D) Probability distributions of content length L of messages gathered by classes of 10 min aggregated volume (circles), and corresponding lognormal fits (dashed curves). For visual clarity an average moving filter of length 10 was applied and only every fifth data point is shown. Fits were performed on the full raw data. (E) Plot of the lognormal fits parameter μ against volume demonstrates the systematic relation between volume and length, dashed line. (F) Plot of the lognormal fits parameter σ^2 against volume.

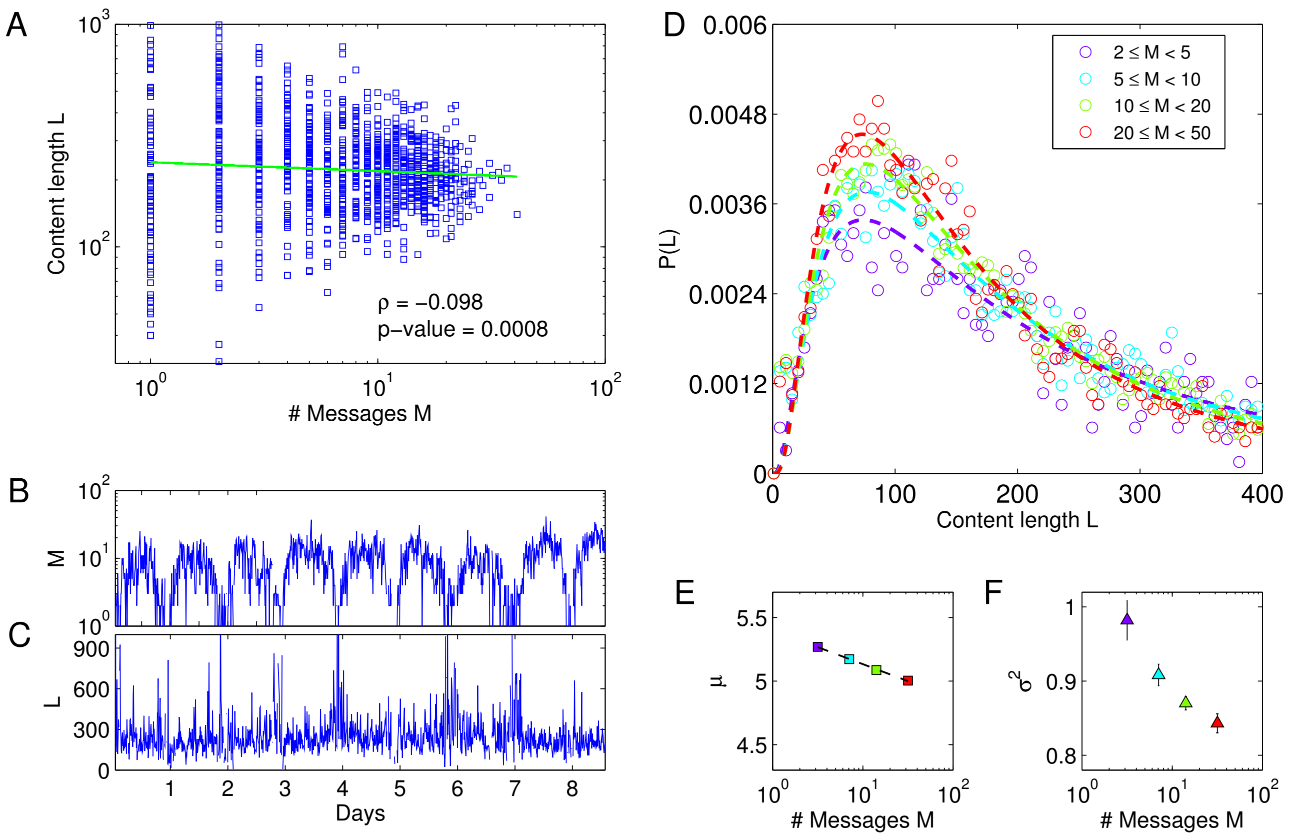

Figure S3. Characteristics of data set 2b: Forum thread one week before the presidential election night. (A) Due to the conversational nature of the thread and the lack of an ongoing important event, volume and content length are much less correlated than in data sets in which events take place. Volume and content length are slightly anti-correlated ($\rho = -0.098$, p-value $= 8 \times 10^{-4}$). (B) Respective hourly volume and (C) content length over time. (D) Probability distributions of content length L of messages gathered by classes of 10 min aggregated volume (circles), and corresponding lognormal fits (dashed curves). For visual clarity an average moving filter of length 10 was applied and only every fifth data point is shown. Fits were performed on the full raw data. (E) Plot of the lognormal fits parameter μ against volume demonstrates the systematic relation between volume and length. (F) Plot of the lognormal fits parameter σ^2 against volume.

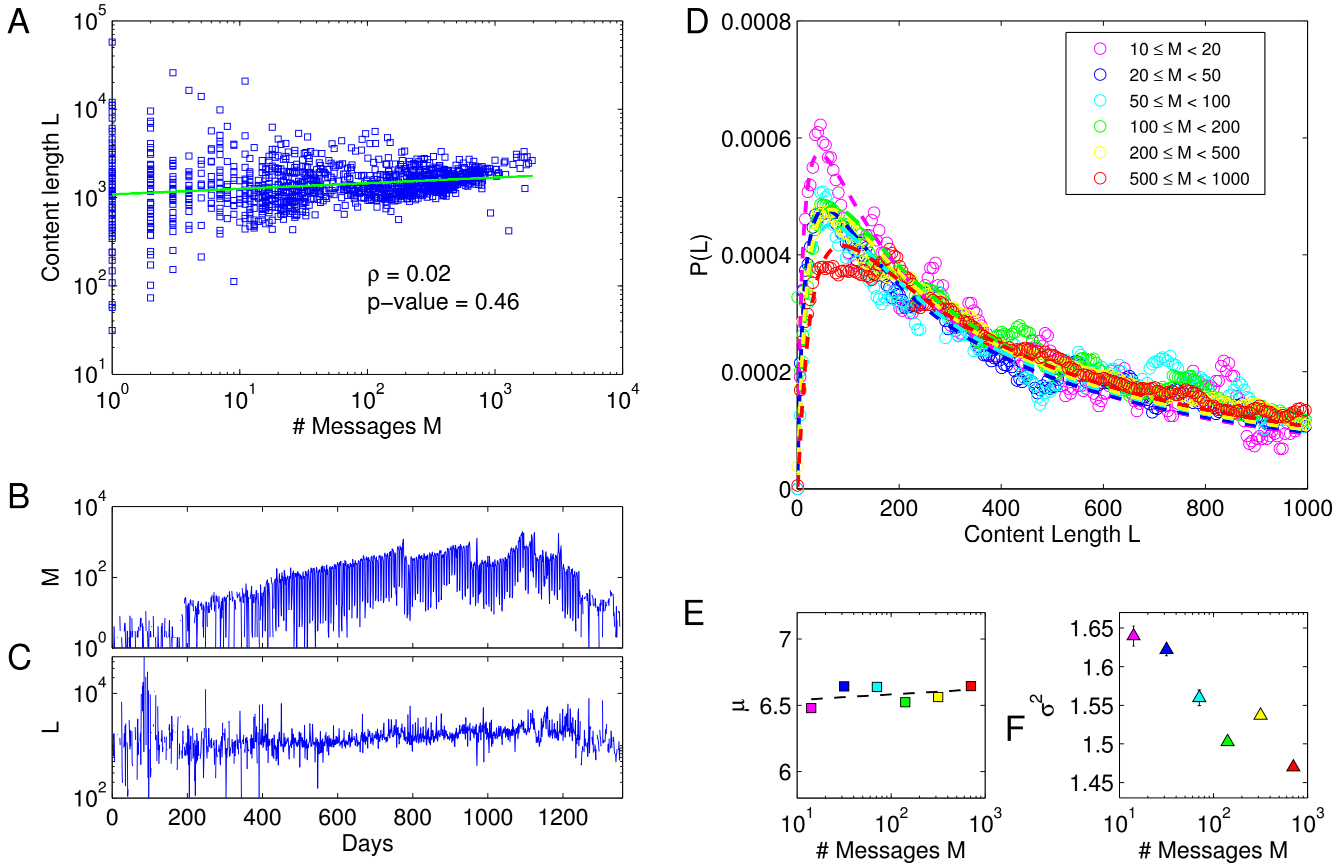

Figure S4. Characteristics of data set 3: Enron email corpus. (A) The microscopic property of content length L (daily average number of characters per message) can be related to the macroscopic property of daily volume (number of messages M) via power law with slope 0.08 (green line). Due to the lack of any specific important event discussed over the emails, volume and content length only present a slight and insignificant correlation. ($\rho = 0.02$, p-value $= 0.46$ for the hypothesis of no correlation). (B) Respective hourly volume and (C) content length over time. (D) Probability distributions of content length L of messages gathered by classes of 10 min aggregated volume (circles), and corresponding lognormal fits (dashed curves). For visual clarity an average moving filter of length 10 was applied and only every fifth data point is shown. Fits were performed on the full raw data. (E) Plot of the lognormal fits parameter μ against volume confirms the absence of any relation between volume and length. (F) Plot of the lognormal fits parameter σ^2 against volume.

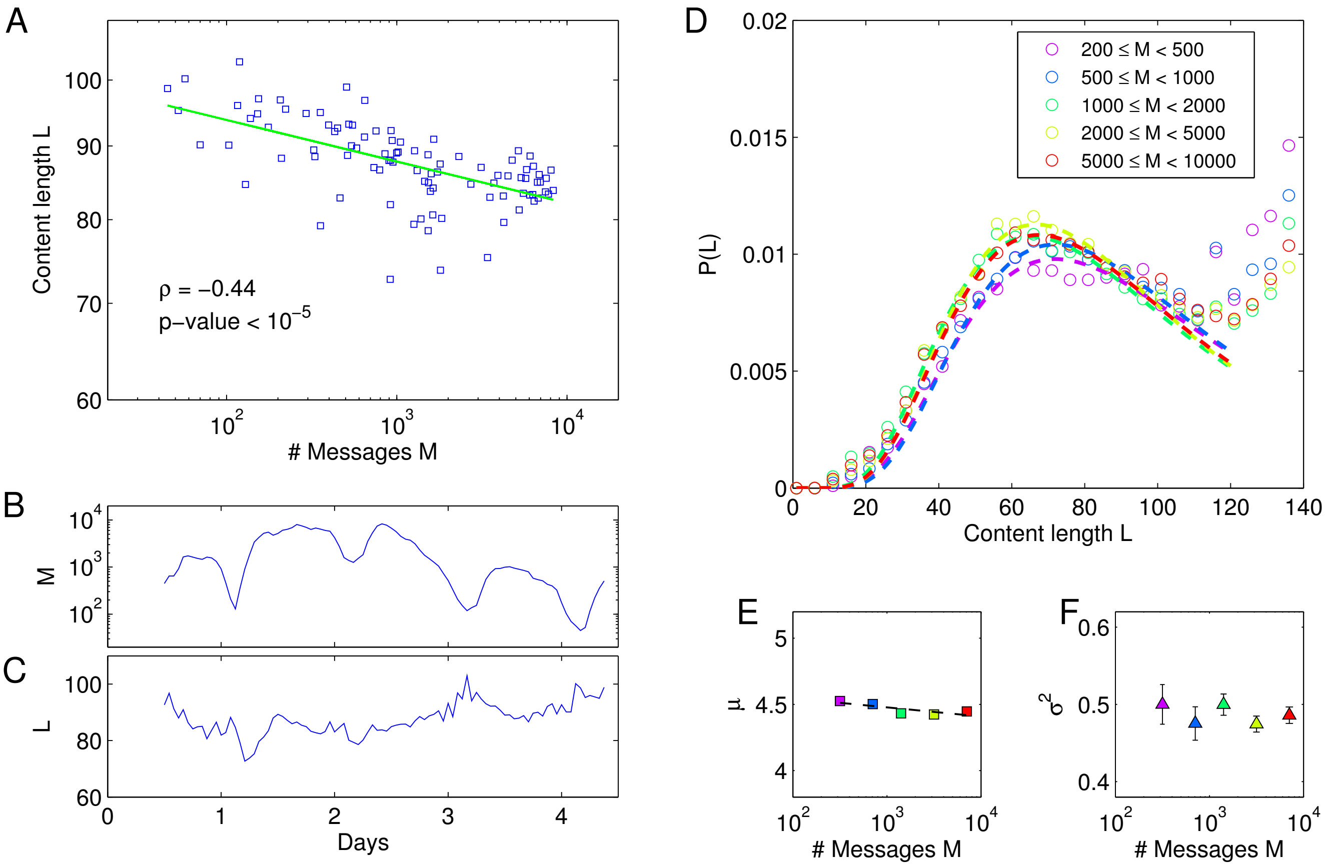

Figure S5. Characteristics of data set 4a: Twitter snow storm. (A) The microscopic property of content length L (average number of characters per message) can be related to the macroscopic property of message rate M via power law with slope -0.03 (green line), a logarithmic fit cannot be rejected as well due to the flat slope. Volume and content length are strongly anti-correlated ($\rho = -0.44$, p-value $< 10^{-5}$ for the hypothesis of no correlation). (B) Respective hourly volume and (C) content length over time. (D) Probability distributions of content length L of messages gathered by classes of hourly volume (circles), and corresponding lognormal fits (dashed curves, fit ranges 0 to 120). For visual clarity an average moving filter of length 5 was applied and only every fifth data point is shown. Fits were performed on the full raw data. (E) Plot of the lognormal fits parameter μ against volume demonstrates the systematic relation between volume and length, dashed line. (F) Plot of the lognormal fits parameter σ^2 against volume.

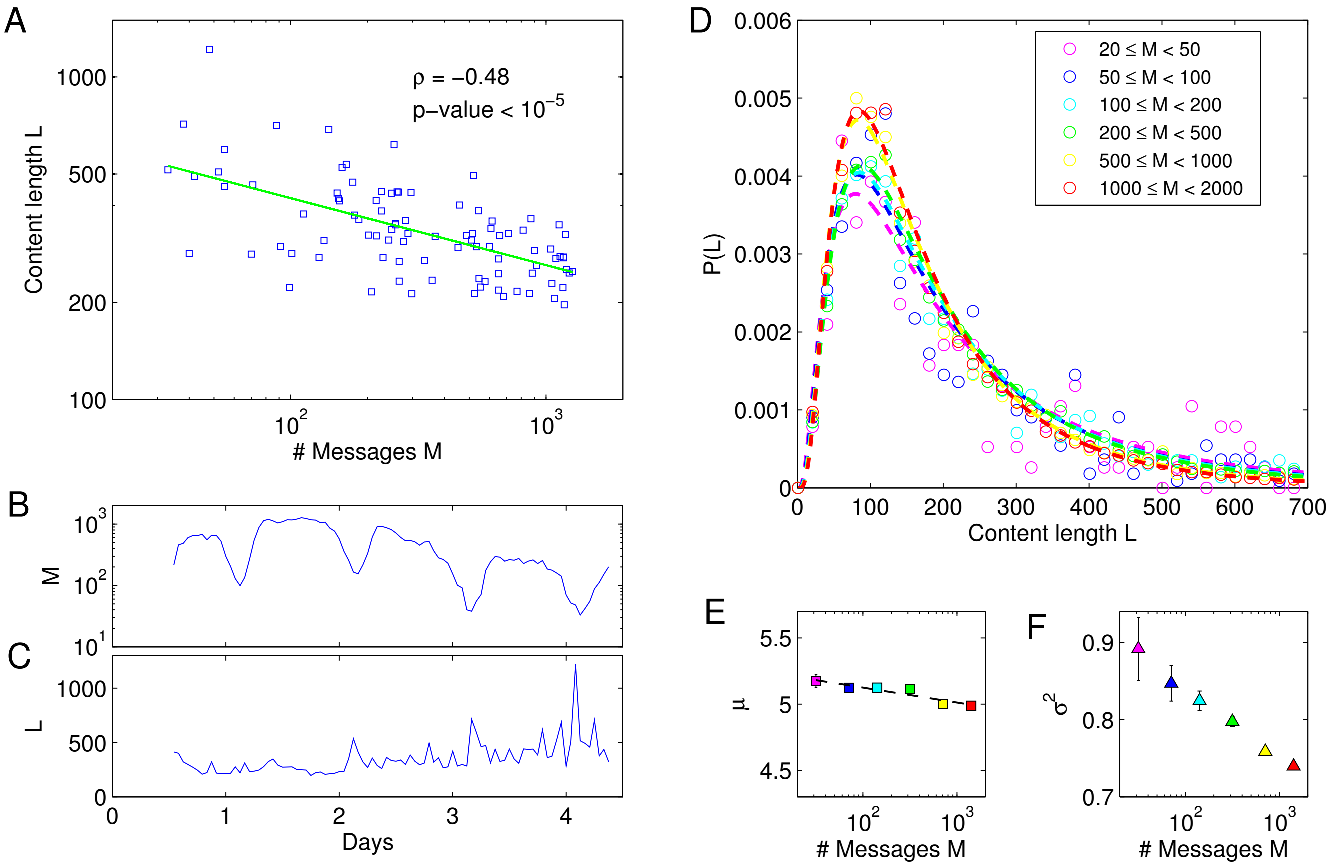

Figure S6. Characteristics of data set 4b: Facebook snow storm. (A) The microscopic property of content length L (average number of characters per message) can be related to the macroscopic property of message rate M via power law with slope -0.21 (green line), a logarithmic fit cannot be rejected as well due to the flat slope. Volume and content length are strongly anti-correlated ($\rho = -0.48$, p-value $< 10^{-5}$ for the hypothesis of no correlation). (B) Respective hourly volume and (C) content length over time. (D) Probability distributions of content length L of messages gathered by classes of hourly volume (circles), and corresponding lognormal fits (dashed curves). For visual clarity an average moving filter of length 5 was applied and only every fifth data point is shown. Fits were performed on the full raw data. (E) Plot of the lognormal fits parameter μ against volume demonstrates the systematic relation between volume and length, dashed line. (F) Plot of the lognormal fits parameter σ^2 against volume.

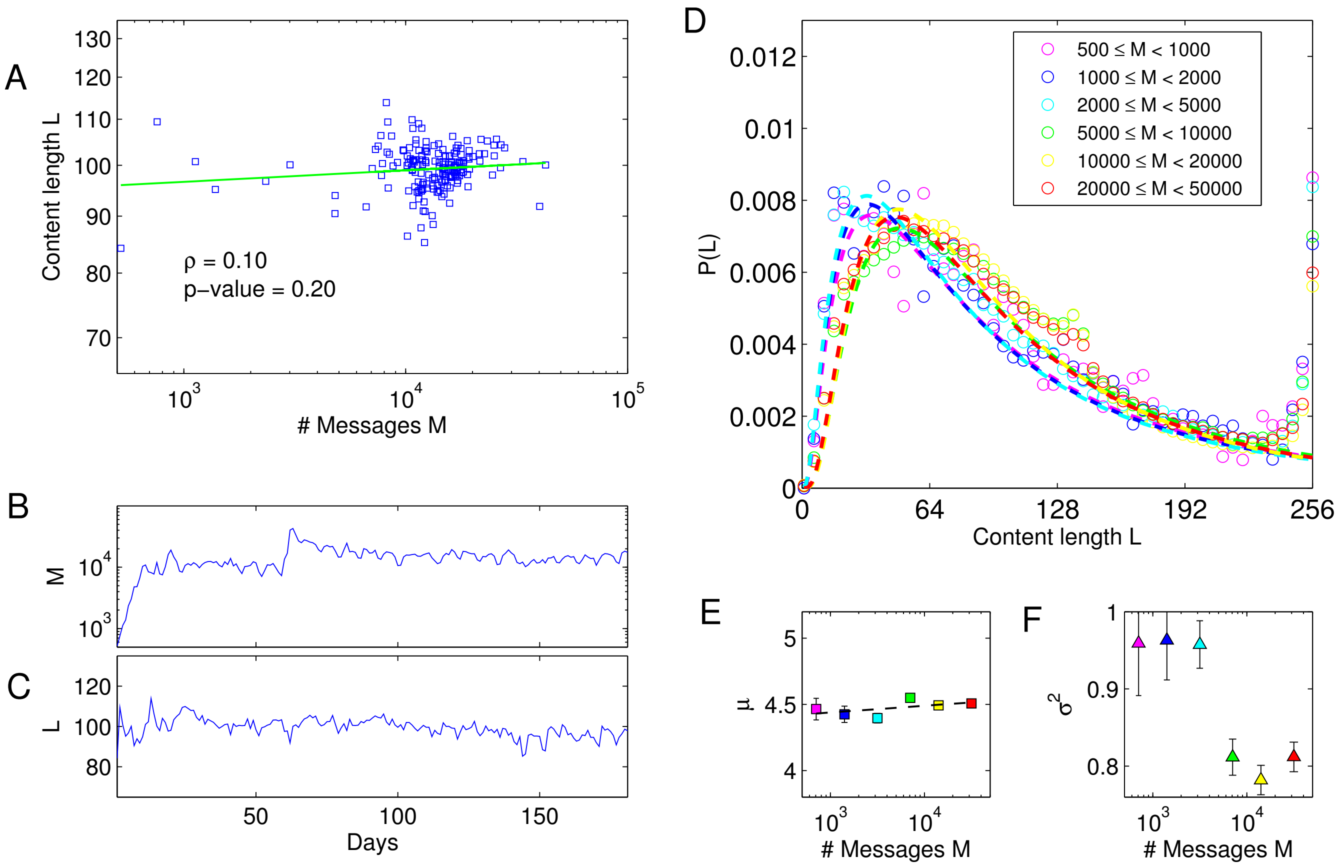

Figure S7. Characteristics of data set 5: App.net. (A) The microscopic property of content length L (average number of characters per message) can be related to the macroscopic property of message rate M via power law with slope 0.01 (green line), a logarithmic fit cannot be rejected as well due to the flat slope. Volume and content length do not present any specific correlation ($\rho = 0.01$, p-value = 0.81 for the hypothesis of no correlation). (B) Respective hourly volume and (C) content length over time. (D) Probability distributions of content length L of messages gathered by classes of 10 min aggregated volume (circles), and corresponding lognormal fits (dashed curves). For visual clarity an average moving filter of length 10 was applied and only every fifth data point is shown. Fits were performed on the full raw data. (E) Plot of the lognormal fits parameter μ against volume confirms the absence of any relation between volume and length. (F) Plot of the lognormal fits parameter σ^2 against volume.

S3. FITTING OF CONTENT LENGTH DISTRIBUTIONS

Apart from Ref. [3], we are not aware of previous distribution analyzes of message lengths, such as of SMS or online messages, however there exists a small number of studies on the distribution of sentence length, usually in the context of Stylometry [4], the statistical study of linguistic style. The lognormal, SI eq. (1), was measured and proposed for sentence lengths at least as far back as 1940 [5] and has been assessed independent of language [6]. An alternative to the lognormal has been suggested later: Sichel [7] has rejected the lognormal for sentence distributions, suggesting an intricate distribution with three parameters arising from a mix of Poisson distributions, SI eq. (2), – now also known as generalized inverse Gaussian or Sichel distribution – yielding better fits compared to the negative binomial, SI eq. (3). The author rejected the use of the lognormal due to lack of discreteness and due to negative skewness after logarithmic transformation observed in a corpus of Greek texts [6]. However, no motivation for the use of the inverse Gaussian nor tests for lognormality were provided. On the other hand, a recent study of Japanese sentences has proposed the lognormal distribution on the basis of a tree-like complexity structure of natural languages [8]. A lognormal sentence length distribution might also be related to the lognormal distribution of speech segment durations [9]. In any case, the distribution of sentence lengths and their origins being relatively understudied leaves the "best" distribution of sentence lengths an open question.

It is reasonable to assume that the distribution of tweet lengths follows the same laws as single sentence distributions, as a tweet is often composed of exactly one sentence. However, for Twitter there exists an arbitrary limitation of 140 characters, possibly distorting the tail. For the other data sets, except for app.net, the messages have no length limitations and can be composed of multiple sentences – for this reason the comparison with literature on single sentence distributions has to proceed with care.

We tested the following distributions which were either suggested in the literature or which might also apply reasonably:

$$f(x;\mu,\sigma) = \frac{1}{x\sigma\sqrt{2\pi}}\, e^{-\frac{(\ln x-\mu)^2}{2\sigma^2}} \text{ (lognormal)} \tag{1}$$

$$f(x;a,b,p) = \frac{(a/b)^{p/2}}{2K_p(\sqrt{ab})} x^{(p-1)} e^{-(ax+b/x)/2} \text{ (Sichel)} \tag{2}$$

$$f(x;r,p) = \frac{\gamma(x+r)}{\gamma(x+1)\gamma(r)}(1-p)^r p^x \text{ (negative binomial)} \tag{3}$$

$$f(x;\mu,\sigma) = \frac{1}{\sigma\sqrt{2\pi}} \mathrm{e}^{-\frac{1}{2}\left(\frac{x-\mu}{\sigma}\right)^2} \text{ (Gaussian)} \tag{4}$$

$$f(x;\sigma) = \frac{x}{\sigma^2} e^{-x^2/2\sigma^2} \text{ (Rayleigh)} \tag{5}$$

$$f(x;k,\theta) = \frac{1}{\theta^k}\frac{1}{\gamma(k)} x^{k-1} e^{-\frac{x}{\theta}} \text{ (gamma)} \tag{6}$$

The K_p in the Sichel distribution is a Bessel function of the second kind. As we show below, measurements are always consistent with lognormals, this distribution cannot be rejected. Other distributions fit as well, so the lognormal is no exclusive candidate. Mean Squared Error (MSE) for the different distributions are given in SI Tables S1 and S2. For a given fit, the MSE is defined as $\mathrm{MSE} = \frac{1}{N-p}\sum_{i=1}^{N} R_i^2$, over the vector of residuals R, where N is the number of observations and p the number of fitted parameters. For all fits the Levenberg-Marquardt algorithm was used.

Data set 1

Before fitting the content length distribution in the Twitter data it has to be noted that the length limitation introduces an artificial elevation of the distribution the closer it comes to the maximum, at which point the distribution peaks. Distributions of content length in the similar microblogging service app.net have shown a similar phenomenon, only less pronounced due to the shifted length limitation to 256 instead of 140 characters [10]. Because of this effect we select the fit range for the Twitter data from 1 to 120, but we cannot rule out the length limitation affecting the distribution also at lower values. For the Twitter data best fits are given by the Sichel distribution, lognormal and negative binomial also perform reasonably well, SI Fig. S8 and SI Table S1. Gaussian, Rayleigh and gamma distributions perform much worse. Comparison with the other data sets shows that in absence of the length limitation better lognormal fits are achieved, see next subsection.

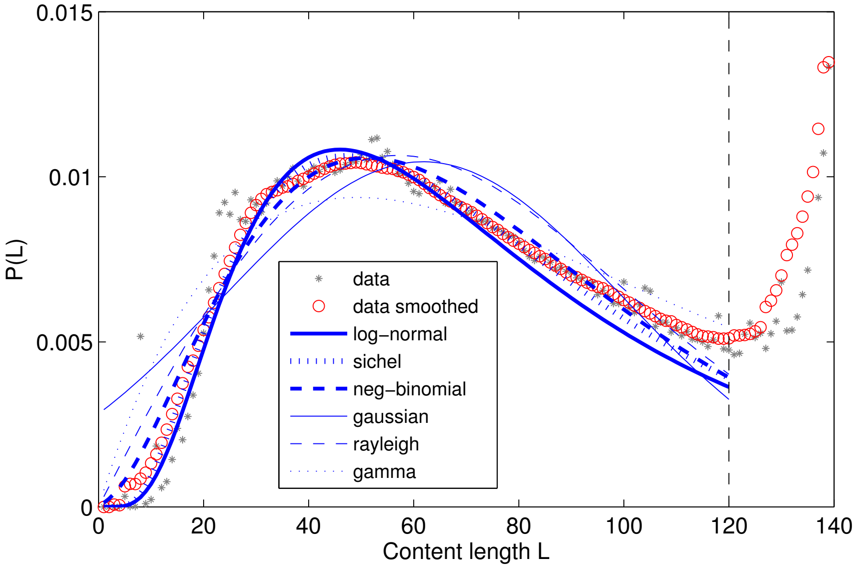

Figure S8. Length distribution of all the tweets in data set 1, and corresponding fits of various distributions. Fit range is 1 to 120. The best fits are given by the Sichel, negative binomial and lognormal distributions. Due to the length limitation of Twitter, the empirical distribution may be artificially elevated already at values also lower than 120. This issue is absent for the other data sets.

Category	lognormal	Sichel	neg. bin.	Gaussian	Rayleigh	gamma
$100 < M < 200$	2.69	2.60	**2.58**	3.11	6.13	4.40
$200 < M < 500$	1.31	1.05	**1.04**	1.45	3.10	2.16
$500 < M < 1000$	1.05	**0.81**	**0.81**	1.53	2.07	2.21
$1000 < M < 2000$	0.88	**0.65**	0.66	1.60	1.26	1.97
$2000 < M < 5000$	1.55	1.23	**1.22**	2.22	1.49	2.35
$5000 < M < 10000$	1.03	**0.85**	1.00	2.88	1.57	2.59
$10000 < M < 20000$	0.94	**0.83**	1.28	3.89	2.21	4.09
$20000 < M < 50000$	2.29	**1.94**	3.54	7.81	5.12	6.30
$50000 < M < 100000$	1.34	**0.98**	2.76	7.38	4.51	6.56
all tweets	0.87	**0.64**	0.85	2.77	1.44	2.04

Table S1. Best fit MSEs ($\times 10^6$) for the tested distributions for the Twitter data set 1. The fit range is 1 to 120. We fitted several samples of the data, where time ranges with different activity levels (measured by the number of tweets) were selected. From the MSEs the Sichel distribution generally fits best. Best fits per category are bolded.

Data set 2a

For the forums data set 2a we do not face the problem of limited lengths and can therefore fit the whole range from 1 to the maximum length of all forum posts of 4266 (we disregarded the first post of length 17,462 due to its special nature of being the introductory post). Fits are shown in SI Fig. S9, corresponding MSEs in SI Table S2. The fits show that all of the following forms fit almost equally well: lognormal, Sichel, negative binomial, gamma. The lognormal however seems to fit the tail best, SI Fig. S10. Note that forum posts contain usually not only one but an arbitrary number of sentences, therefore the comparison with the Sichel distribution in the tail is not perfectly adequate. Gaussian and Rayleigh distributions again perform badly.

Category	lognormal	Sichel	neg. bin.	Gaussian	Rayleigh	gamma
$20 < M < 40$	0.137	**0.133**	0.133	0.158	0.207	0.133
$40 < M < 80$	**0.114**	0.115	0.119	0.158	0.213	0.117
$80 < M < 160$	0.187	0.177	0.176	0.206	0.287	**0.175**
$160 < M < 320$	0.131	0.115	0.114	0.154	0.220	**0.113**
$320 < M < 640$	0.242	0.213	**0.210**	0.249	0.392	0.215
all posts	0.085	**0.083**	0.083	0.122	0.255	0.084

Table S2. Best fit MSEs ($\times 10^6$) for the tested distributions for the forum data set 2a. The fit range is 1 to 4266. We fitted several samples of the data, where time ranges with different activity levels (measured by the number of messages) were selected. From the MSE all of the following forms fit almost equally well: lognormal, Sichel, negative binomial, gamma. Best fits per category are bolded.

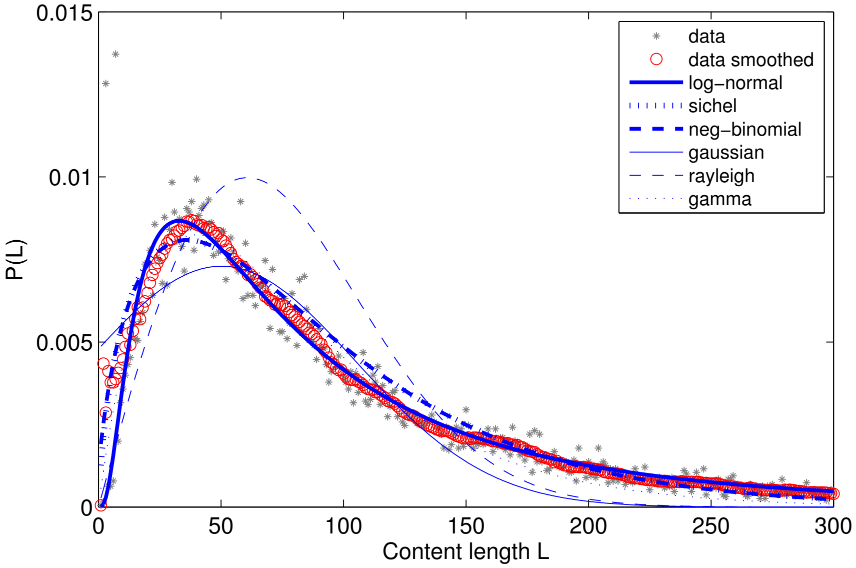

Figure S9. Length distribution of all the posts in data set 2a, and corresponding fits of various distributions. Fit range is 0 to 4266. Again all of the following forms fit almost equally well: lognormal, Sichel, negative binomial, gamma. Smoothed data (using a moving average filter of length 20) is displayed by red circles.

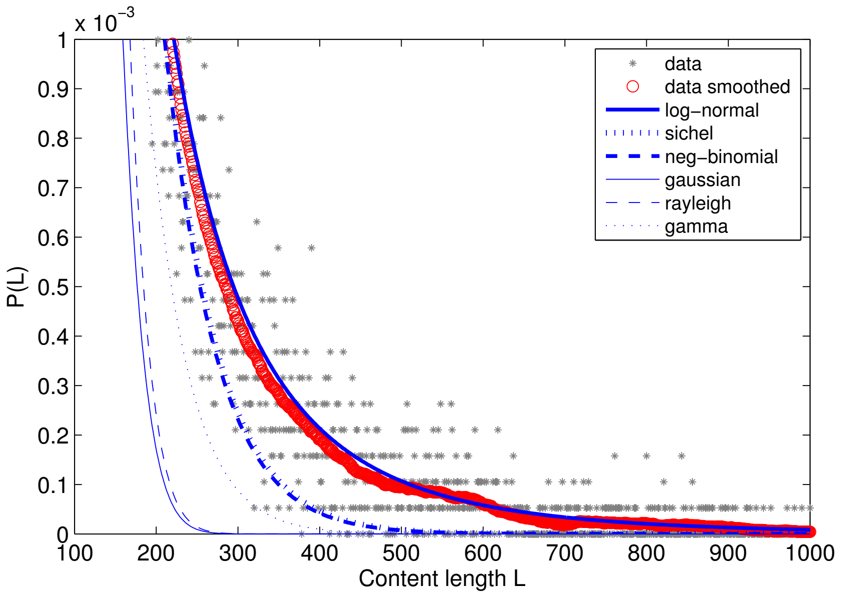

Figure S10. Detail from Fig. S9 showing the tail of the distribution. Raw data points are shown in in grey, the smoothed data (using a moving average filter of length 100) in red. Out of the considered distributions the lognormal fits the tail best visually.

S4. INFLUENCE OF DIFFERENT PARAMETERS

The length distributions shown in the main text, Fig. 2, are based on all tweets of dataset 1, and reflect the collective behavior of a heterogeneous collection of users. Users composes content on their own free will depending on their interest in the golf event, on their exposure to the event (through TV, radio, friends tweets, ...), on their specific intentions when tweeting (just making a comment, communicating with friends, maintaining their fans base ...) and certainly other factors. The point of this section is to check whether our findings could result from some bias caused by specific users or usage of Twitter. To this end, we selected subsets of tweets with some characteristics within data set 1 and compare the obtained results with all this subsets. Fig. 4 in the main text shows the length distributions obtained by selecting tweets responding to some criteria, while Fig. S11 shows the variation of the lognormal fit parameters with the volume. More specifically,

- the first column highlights the difference between tweets without mentions ($\sim 80\%$ of the dataset) and tweets with mentions. The impact of message rate (or volume) appears significantly less strong for tweets with mentions. Indeed, as the number of mentions increases, the disparity between the length distributions of the different volume classes decreases and so does the dependance of $\mu = \langle \log L \rangle$ with volume M. This can be understood as different underlying motives for writing a message: while users mentioning each other are more concerned about communicating with each other, users who don't make any mention are more likely to display a pure reaction to the event. Notice also how the starting point of the distributions are shifted to the right when the number of mentions increases, which reflects a mention's average length.

- the second column shows the influence of number of hashtags. Putting aside tweets with 4 hashtags (a somehow extreme case concerning this amounts to less than 3% of the dataset), the number of hashtags mainly shift the distribution to the right, accounting for a typical hashtag length. The plots of μ *vs* M appears almost identical up to a translation. Notice also that due to the 140 characters limitation, the distributions become more narrow as the number of hashtags increases, as reflected by the the decrease of the fits variance parameter σ^2.

- the third column displays the effect of the total number of tweets (in the data set) written by a user. The most notable detail is that the volume has no effect on the length distributions of tweets written by the most active users (who wrote more than 100 tweets during the golf event). An exploration of the dataset indicates that these tweets mostly correspond to automatic messages written by bots or advertising tweets. The translation in the μ *vs* $\log M$ curve of Fig. S11 when the total number of tweets increase from 1 to 100 also seems to indicate that people who use Twitter more often make significantly longer tweets. This might be a sign that they are more at ease with the 140 characters limitation.

- the fourth column differentiates the users by their connectivity to other users by their number of followees (i.e., people whose tweets they are reading). While the statistics appears insufficient to validate the fits for the few tweets written by users with more than 10^4 followees, the number of follows does not appear to generate specific reaction to the golf event.

- the fifth column differentiates the users by their connectivity to other users by number of followers (i.e., people who read their tweets). While the length distributions always strongly depend on the volume, the minority ($\sim 8\%$) of users with a lot of followers (more than 10^3) tends to write significantly longer tweets, as it clearly appears on Fig. S11. This may reflect an underlying motive of self-promotion of these specific users, who try to satisfy their audience with more elaborated messages.

- finally, the sixth column sheds light on the influence of the users' smallest hourly message rate M_0 in which they tweet (see Fig. 4 in the main text). The behavior of users with a very low M_0 does not seems to be affected by the volume. Digging into the data allowed us to identify these users as a group of people using some of the tracked hashtags (as it happens, mostly '#golf') used to build the dataset, while talking about a subject completely unrelated to the golf masters (as it happens, car brands). These specific users are identified through their M_0 value because they continue to tweet after the golf event, when the number of tweets per hour goes down. Otherwise, the length distributions are not significantly affected by the M_0 value of the users. This result ensures that the message rate dependency of the distributions is not due to a bias introduced by the numerous users who tweet only once or twice during the high-volume times.

Based on all these observation, one can attempt to built an 'ideal' subset by selecting only tweets with 0 mentions (hence removing 'chit-chatting' users), 1 hashtag (to avoid shifting issues), written by users with less than 100 tweets

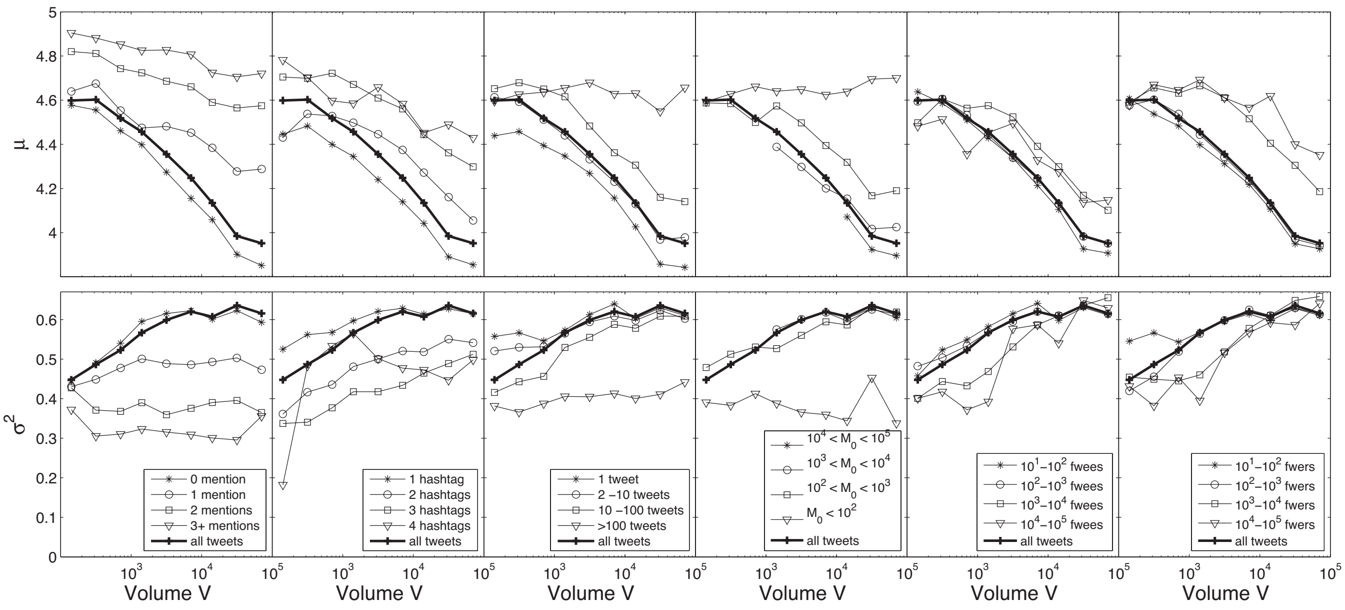

Figure S11. Dependence of the lognormal fitted parameters with the volume. The bold line correspond to the fit parameters obtained when using all the tweets. As explained in the previous section, all fits are based on the distributions values between 1 and 120 characters.

(hence removing bots and ads), less than 1000 followees or followers (hence removing self-promoting users) and a volume threshold M_0 of at least 100. This filtered subset contains $\sim 50\%$ of the original data set. We present the analysis of this 'ideal' subset in Fig S12. Notice in particular the approximately linear dependency of fit parameter μ with $\log M$ and the independence of the fit parameter σ^2 with $\log M$.

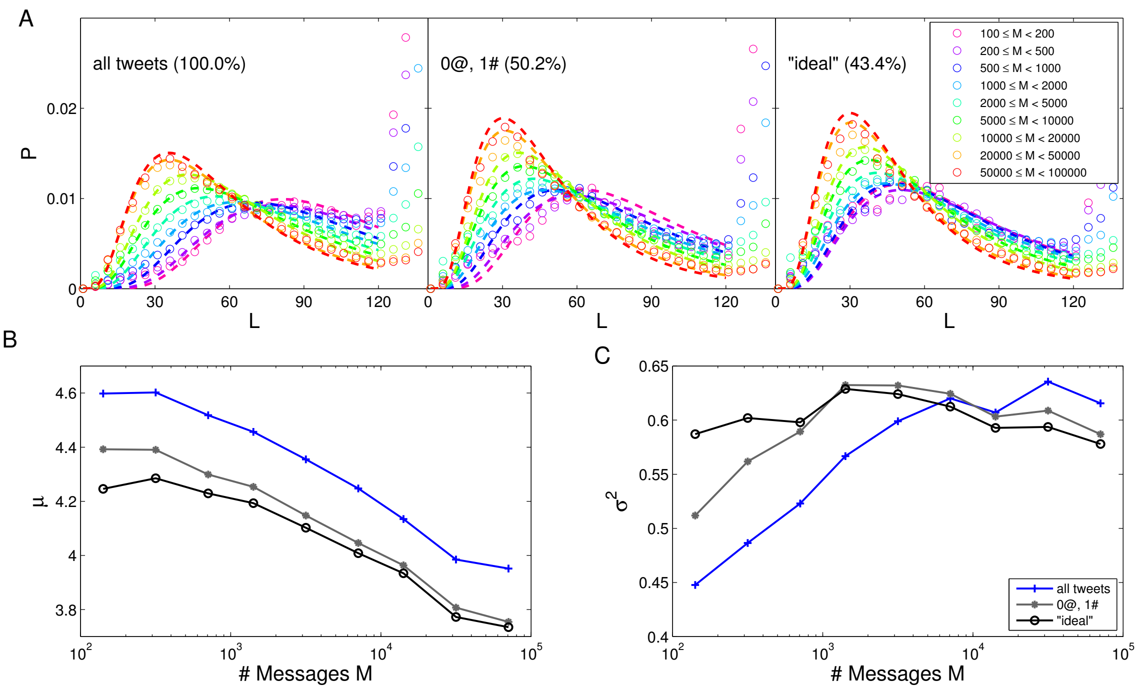

Figure S12. Analysis of the 'ideal' subset of tweets. (A) Probability distributions of content length L of messages gathered by classes of hourly volume (circles), and corresponding lognormal fits (dashed curves). For visual clarity an average moving filter of length 5 was applied and only every fifth data point is shown. (B) Plot of the lognormal fits parameter μ against volume M demonstrates the systematic relation between volume and length, dashed line. (C) Plot of the lognormal fits parameter σ^2 against volume.

S5. INFLUENCE OF TIME STEP

The results presented in the main text are based on an hourly partitioning of data set 1. In this section, we check that our results are not substantially affected by the time step used in the analysis, thus assuring robustness of our findings. Indeed, Figs. S13 and S14 display a reiteration of the analysis presented in the main text, but for 5 min and 3 hours time windows, respectively. The results are consistent with our 1-hour time step findings: First, anti-correlation between volume M and average length L at the macroscopic level (log log relation with coefficient -0.077 ± 0.002). Second, log-normal distribution of tweet length, with $\mu \equiv < \log L >$ displaying a linear dependency on $\log M$, and $\sigma^2 \equiv \Delta(\log L)^2$ independent of M for high volumes. Results are thus robust on a range of time steps, see also SI Table S3. Of course excessively diverging time steps become intractable: if too short, time spans will contain a too small number of tweets (in the extreme case only either 1 or 0 tweets), if too long, data becomes too smoothed and important local variations vanish.

timestep	ρ	p-value	α	β
5min	-0.46	3.7×10^{-74}	-0.070	-0.121
10min	-0.51	2.4×10^{-46}	-0.073	-0.117
1h	-0.62	1.7×10^{-13}	-0.077	-0.117
3h	-0.70	6.1×10^{-7}	-0.076	-0.109

Table S3. Characteristic parameters measured on data set 1 for different timesteps. The anti-correlation ρ and its significance level change due to the different number of data points, but the exponents α and β stay at a similar level.

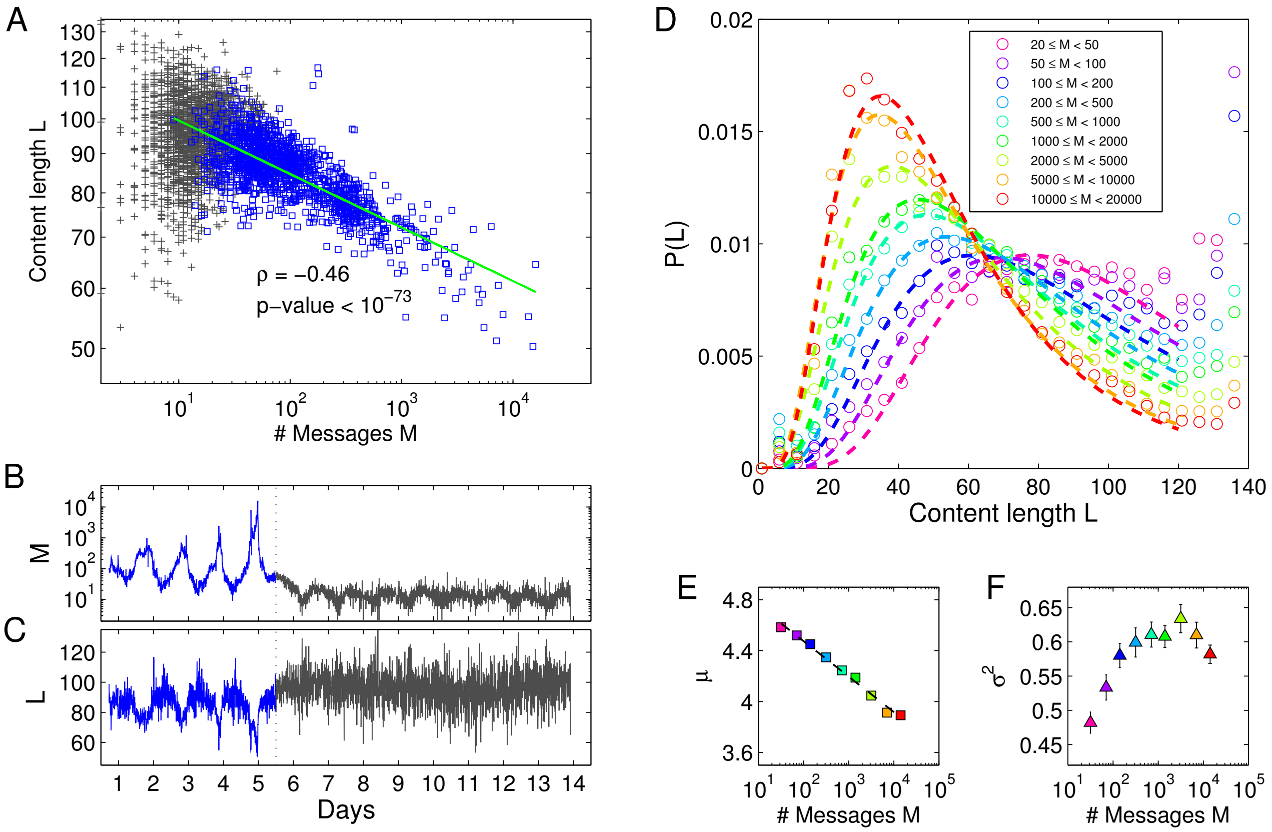

Figure S13. Characteristic of data set 1 with a 5 min timestep. (A) The microscopic property of content length L (average number of characters per message) can be related to the macroscopic property of volume (number of messages M) via power law with slope -0.070 (green line), a logarithmic fit cannot be rejected as well due to the flat slope. Blue squares are average values on 3 hours time windows from the first five days in which the Masters Tournament took place, grey crosses are the average values from subsequent times. Volume and content length are strongly anti-correlated ($\rho = -0.46$, p-value $< 10^{73}$ for the hypothesis of no correlation) but not afterwards ($\rho = 0.07$, p-value $= 0.002$). (B) Respective volume and (C) content length over time. (D) Probability distributions of content length L of messages gathered by classes of 5 min aggregated volume (circles), and corresponding lognormal fits (dashed curves, fit ranges 0 to 120). For visual clarity an average moving filter of length 5 was applied and only every fifth data point is shown. Fits were performed on the full raw data. (E) Plot of the lognormal fits parameter μ against volume demonstrates the systematic relation between volume and length, dashed line.(F) Plot of the lognormal fits parameter σ^2 against volume.

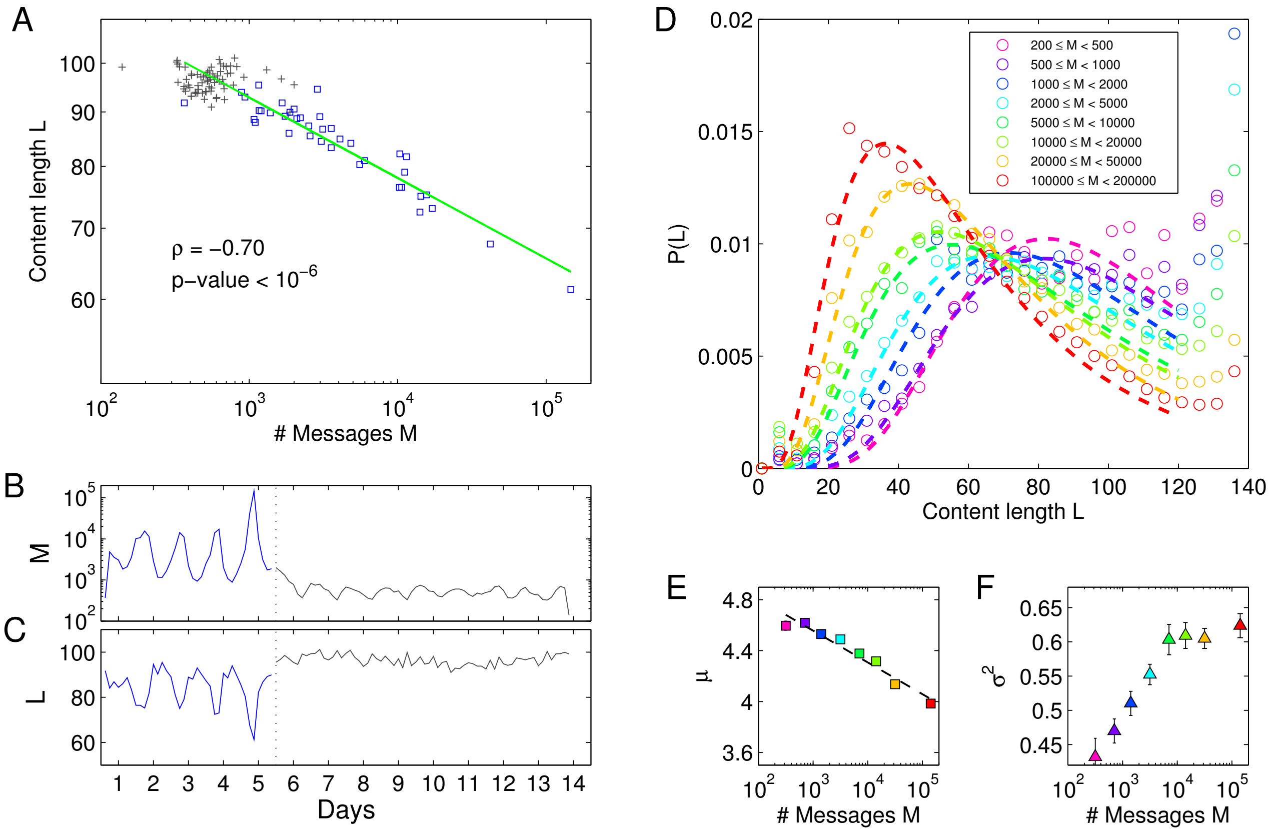

Figure S14. Characteristic of data set 1 with a 3 hours timestep. (A) The microscopic property of content length L (average number of characters per message) can be related to the macroscopic property of volume (number of messages M) via power law with slope -0.076 (green line), a logarithmic fit cannot be rejected as well due to the flat slope. Blue squares are average values on 3 hours time windows from the first five days in which the Masters Tournament took place, grey crosses are the average values from subsequent times. Volume and content length are strongly anti-correlated ($\rho = -0.70$, p-value $< 10^6$ for the hypothesis of no correlation) but not afterwards ($\rho = -0.10$, p-value $= 0.39$). (B) Respective volume and (C) content length over time. (D) Probability distributions of content length L of messages gathered by classes of 3 hours aggregated volume (circles), and corresponding lognormal fits (dashed curves, fit ranges 0 to 120). For visual clarity an average moving filter of length 5 was applied and only every fifth data point is shown. Fits were performed on the full raw data. (E) Plot of the lognormal fits parameter μ against volume demonstrates the systematic relation between volume and length, dashed line.(F) Plot of the lognormal fits parameter σ^2 against volume.

[1] González-Bailón S, Wang N, Rivero A, Borge-Holthoefer J, Moreno Y (2012) Assessing the bias in communication networks sampled from twitter. Available at SSRN 2185134 .

[2] Shetty J, Adibi J (2004) The enron email dataset database schema and brief statistical report. Information Sciences Institute Technical Report, University of Southern California 4.

[3] Sobkowicz P, Thelwall M, Buckley K, Paltaglou A Gand Sobkowicz (2013) Lognormal distributions of user post lengths in internet discussions - a consequence of the weber-fechner law? EPJ Data Science 2.

[4] Holmes D (1985) The analysis of literary style–a review. Journal of the Royal Statistical Society Series A (General) : 328–341.

[5] Williams C (1940) A note on the statistical analysis of sentence-length as a criterion of literary style. Biometrika 31: 356–361.

[6] Wake W (1957) Sentence-length distributions of Greek authors. Journal of the Royal Statistical Society Series A (General) 120: 331–346.

[7] Sichel H (1974) On a distribution representing sentence-length in written prose. Journal of the Royal Statistical Society Series A (General) : 25–34.

[8] Furuhashi S, Hayakawa Y (2012) Lognormality of the distribution of japanese sentence lengths. Journal of the Physical Society of Japan 81: 034004.

[9] Rosen K (2005) Analysis of speech segment duration with the lognormal distribution: A basis for unification and comparison. Journal of Phonetics 33: 411–426.

[10] (retrieved 2013-01-29). http://diegobasch.com/first-month-on-app-net-charts-and-stats.
